# TWIST1 Plays Role in Expression of Stemness State Markers in ESCC

**DOI:** 10.3390/genes13122369

**Published:** 2022-12-15

**Authors:** Mohammad Hossein Izadpanah, Mohammad Mahdi Forghanifard

**Affiliations:** 1 Division of Human Genetics, Immunology Research Center, Avicenna Research Institute, Mashhad University of Medical Sciences, Mashhad 9196773117, Iran; 2 Department of Biology, Damghan Branch, Islamic Azad University, Damghan 3671637849, Iran

**Keywords:** TWIST1, stem cell marker, cancer stem cells (CSCs), self-renewal, ESCC, EMT

## Abstract

Background: Stemness markers play critical roles in the maintenance of key properties of embryonic stem cells (ESCs), including the pluripotency, stemness state, and self-renewal capacities, as well as cell fate decision. Some of these features are present in cancer stem cells (CSCs). TWIST1, as a bHLH transcription factor oncogene, is involved in the epithelial–mesenchymal transition (EMT) process in both embryonic and cancer development. Our aim in this study was to investigate the functional correlation between *TWIST1* and the involved genes in the process of CSCs self-renewal in human esophageal squamous cell carcinoma (ESCC) line KYSE-30. Methods: *TWIST1* overexpression was enforced in the ESCC KYSE-30 cells using retroviral vector containing the specific pruf-IRES-GFP-hTWIST1 sequence. Following RNA extraction and cDNA synthesis, the mRNA expression profile of *TWIST1* and the stem cell markers, including *BMI1*, *CRIPTO1*, *DPPA2*, *KLF4*, *SOX2*, *NANOG,* and *MSI1,* were assessed using relative comparative real-time PCR. Results: Ectopic expression of *TWIST1* in KYSE-30 cells resulted in an increased expression of *TWIST1* compared to control GFP cells by nearly 9-fold. Transduction of *TWIST1*-retroviral particles caused a significant enhancement in *BMI1*, *CRIPTO1*, *DPPA2*, *KLF4,* and *SOX2* mRNA expression, approximately 4.5-, 3.2-, 5.5-, 3.5-, and 3.7-folds, respectively, whereas this increased *TWIST1* expression caused no change in the mRNA expression of *NANOG* and *MSI1* genes. Conclusions: *TWIST1* gene ectopic expression in KYSE-30 cells enhanced the level of cancer stem cell markers’ mRNA expression. These results may emphasize the role of *TWIST1* in the self-renewal process and may corroborate the involvement of *TWIST1* in the stemness state capacity of ESCC cell line KYSE-30, as well as its potential as a therapeutic target.

## 1. Introduction

Tumor progression and metastasis is a complicated process, during which the primary tumor cell population is able to escape and survive in the circulation and invade and proliferate in another microenvironment [1]. Epithelial–mesenchymal transition (EMT) and cancer stem cells (CSCs) are considered two critical subjects in cancer metastasis. Identification of the molecular mechanisms behind EMT and CSC formation can provide impressive insights to prevent tumor metastasis and introduce therapeutic targets [2]. EMT, as a cellular–physiological process with reversible alteration, correlates with stemness properties and develops cancer-related changes in the cell, including invasiveness, decreased cellular adhesion and polarity, and increased motility and invasion, as well as resistance to apoptosis [3,4]. These adaptive alterations can exist between EMT and its reverse process (mesenchymal–epithelial transition; MET) in epithelial malignancies [5]. EMT and metastasis are induced through the activation of different growth factors (such as HGF, PDGF, IGF, EGF, and FGF); extracellular matrix (ECM); numerous zinc-finger transcription factors (TFs), including TWIST1, ZEB, SLUG, E47, SIP1, and SNAIL families; and a variety of cell signaling pathways, including Wnt, Notch, TFGβ, NF-κB, integrins, and tyrosine-kinase receptors [1,6]. The dysregulation of these factors plays a key role in the inhibition of cell polarity through the downregulation of epithelial cell markers, such as E-cadherin and cytokeratin 18, and the upregulation of mesenchymal cell markers, such as N-cadherin, fibronectin, and vimentin, as the EMT hallmarks [7]. The EMT process can generates CSC phenotypes in tumor cell populations that acquire self-renewal properties, proliferation and differentiation potential, expanding ability within tumor, and drug resistance [7,8]. There is a closely reciprocal biological link between EMT and the CSC state [9]. A better understanding of the relation between EMT and CSCs in malignancies can help to recognize the complicated signaling pathways controlling the CSCs/EMT axis during the formation of tumor bulk and residual cells responsible for relapse and metastasis, tumor progression, and targeted therapeutic responses [9]. The molecular mechanisms behind EMT program and CSC phenotype consists of alterations in both the intracellular signaling pathways and the extracellular secreted proteins of tumor cells [8]. The self-renewal and differentiation process in CSCs are mainly regulated through transcriptional regulators (such as OCT4, Nanog, YAP/TAZ, and Myc), as well as signaling pathways (such as Wnt, Notch, HH, TGF-β, PI3K/Akt, EGFR, and JAK/STAT), thereby being able to produce, expand, and maintain more CSCs populations, as well as all the non-CSC progeny in a tumor [10,11].

TWIST1, a basic helix-loop-helix transcription factor, is a key mediator of the mesenchymal/CSC state and can link EMT to self-renewal [4,12]. The upregulation of TWIST1 leads to the activation of the antiapoptotic process during EMT development, increased cell angiogenesis/invasion/migration/metastatic potential, disassembly of cell adhesion, CSC-like features maintenance, and restriction of cellular differentiation, as well as patients’ poor prognosis and survival rate in epithelial cancers [13].

Hence, the evaluation of the EMT mechanism in CSCs regulation is an essential need to identify new approaches for therapeutic strategies preventing cancer progression and improving the prognosis of the disease [6]. Our aim in this study was to evaluate the impact of *TWIST1* ectopic expression on the expression pattern of stemness and self-renewal-associated genes, including *BMI1*, *CRIPTO1*, *DPPA2*, *KLF4*, *SOX2*, *NANOG,* and *MSI1* in ESCC cell line KYSE-30, to elucidate possible crosstalk between *TWIST1* and the self-renewal state of the cells.

## 2. Materials and methods

### 2.1. In Silico Sequence Analysis

*BMI1*, *CRIPTO1*, *DPPA2*, *KLF4,* and *SOX2* mRNAs and gene sequences were obtained from Genebank (accession numbers NM_005180.9, NM_001174136, NM_138815, NM_004235.6, and NM_004235, respectively). The sequence analysis was performed by using CLC Main Workbench version 20 (CLC bio).

### 2.2. Cell Lines and Culture Condition

GP293T (a HEK293T-derived retroviral packaging cell line) and the human ESCC (KYSE-30) cell line were obtained from the Pasteur Institute Cell Bank of Iran (http://en.pasteur.ac.ir/). These cells were cultured in Dulbecco’s modified Eagle’s medium (DMEM; Biosera, Shanghai, China) and RPMI 1640 medium (Biosera), respectively, supplemented with 100 U/mL penicillin–streptomycin (Gibco, FL, USA) and 10 % heat-inactivated fetal bovine serum (FBS; Gibco, FL, USA) in a humidified atmosphere at 37 °C with 5% CO_2_.

### 2.3. Retroviral Production and Enforced TWIST1 Overexpression

The Pruf-IRES-GFP-hTWIST1 and control Pruf-IRES-GFP plasmids were obtained for generating retroviral particles, as described before [12,14]. Briefly, plasmids of Pruf-IRES-GFP-hTWIST1, pGP, and pMD2 were co-transfected in GP293T cells according to the standard calcium phosphate precipitation method. Viral particles were harvested 24 and 48 h after transfection and resuspended in fresh medium. Eventually, KYSE-30 cells were transduced with recombinant retroviral particles of Pruf-IRES-GFP (control) and Pruf-IRES-GFP-hTWIST1 (1 × 10^5^ cells/6-well plate). Retroviral transduction was performed twice and evaluated through inverted fluorescence microscopy.

### 2.4. RNA Extraction, cDNA Synthesis, Comparative Real-Time PCR, and Statistical Analysis

The RNA extraction from the Pruf-IRES-GFP- and Pruf-IRES-GFP-hTWIST1-transduced ESCC cell line, DNase I treatment, cDNA synthesis, and quantitative RT-PCR expression analysis have been described in detail previously [12,15,16]. The mRNA expression profiles of the involved genes in the process of cancer stem cell self-renewal, including *BMI1*, *CRIPTO1*, *DPPA2*, *KLF4*, *SOX2*, *NANOG,* and *MSI1,* were evaluated by specific primer sets demonstrated in Table 1.

## 3. Results

### 3.1. Sequence Analysis of Cancer Stem Cell Self-Renewal Genes Promoter

**Figure 1:**
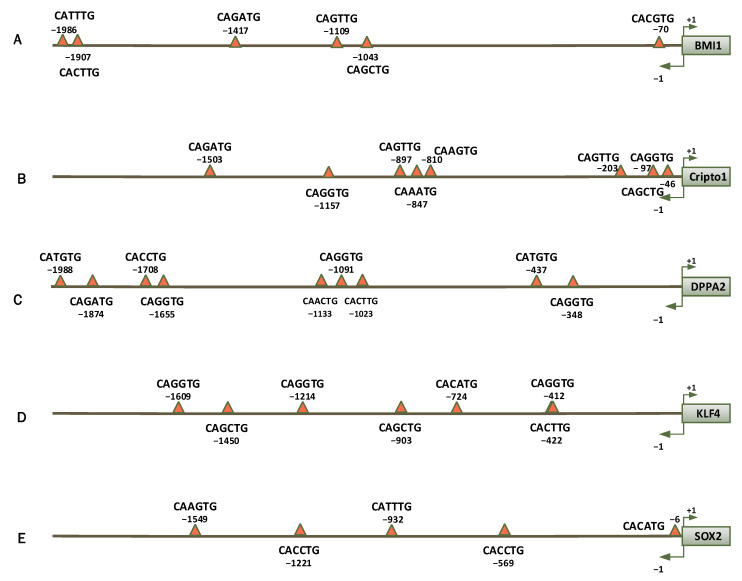
Schematic view of the positions and sequences of seven E-box hexanucleotide consensus sequence CANNTG within 2 Kb upstream of the genes transcription start site. (**A**) *BMI1* promoter, (**B**) *Cripto1* promoter. (**C**) *DPPA2* promoter. (**D**) *KLF4* promoter. (**E**) *SOX2* promoter.

Transcription unit and its upstream region sequences of the *BMI1, CRIPTO1, DPPA2, KLF4,* and *SOX2* genes were evaluated to check and find the probable E-boxes. Several different E-boxes were found in the −2 kb upstream region of these genes before the starting site. Interestingly, some of the E-boxes were located close to the transcription start site in position −70 for BMI1; −46, −97 and −203 for *CRIPTO1*; in −348 and −437 for *DPPA2*; in− 412 and −422 for *KLF4;* and finally in −1 and −6 for *SOX2*. Other E-boxes were highlighted in Figure 1. Furthermore, there were several E-boxes in the *CSCs* gene transcription units located in both exon and intron regions. (Table 2, Table 3, Table 4, Table 5 and Table 6).

### 3.2. Upregulation of TWIST1 in ESCC Cell Line KYSE-30

The expression of *TWIST1* was investigated in Pruf-IRES-GFP-hTWIST1 in comparison with Pruf-IRES-GFP-control KYSE-30 cells to confirm the increased expression of *TWIST1*. The fluorescent microscopy confirmed the efficiency of transduction in Pruf-IRES-GFP-hTWIST1 and Pruf-IRES-GFP control KYSE-30 cells, as revealed before [16]. The significant overexpression by nearly 9-fold (log2 fold change) of *TWIST1* was detected in retroviral Pruf-IRES-GFP-hTWIST1-transduced cells compared to control.

### 3.3. Ectopic Expression of TWIST1 Increased Expression of Cancer Stem Cell Self-Renewal Genes

**Figure 2:**
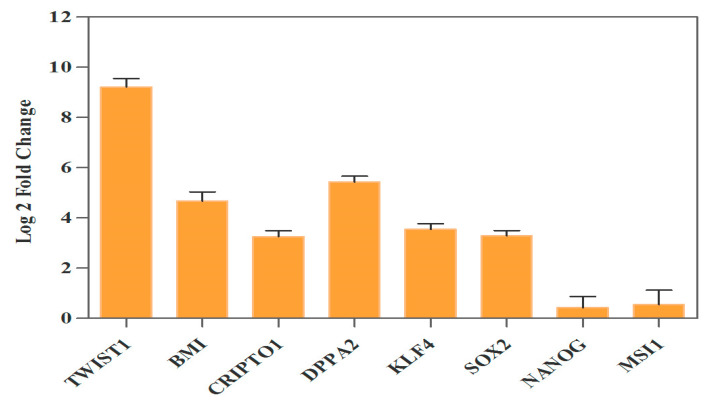
Ectopic expression of *TWIST1* gene has a significant impact on cancer stem cell self-renewal genes’ expression in KYSE-30 cells. Retroviral transduction enforced significant *TWIST1* overexpression in pruf-IRES-GFP-hTWIST1 by nearly 9-fold compared to GFP control cells, causing a 4.5-, 3.2-, 5.5-, 3.5-, and 3.7-fold increase in the mRNA level of *BMI1*, *CRIPTO1*, *DPPA2*, *KLF4*, and *SOX2*. Forced expression of *TWIST1* had no effect on the mRNA expression of *NANOG* and *MSI1* genes.

The stable enforced high expression of *TWIST1* in the ESCC cell line increased the mRNA expression of selected candidate CSC genes. After confirming the *TWIST1* gene overexpression in KYSE-30 cells, we assessed the mRNA expression profile of specific cancer stem cell self-renewal genes consequently. The upregulation of *TWIST1* led to a significant increase in the levels of *BMI1*, *CRIPTO1*, *DPPA2*, *KLF4,* and *SOX2* mRNA expression (4.5-, 3.2-, 5.5-, 3.5-, and 3.7-log 2 fold change, respectively), while *TWIST1* overexpression had no effect on the mRNA expression of *NANOG* and *MSI1* genes. Data are represented in Figure 2.

## 4. Discussion

The EMT process and stemness state are tightly linked together. The common shared regulators and signaling pathways in these events can assist in a better understanding of the connection between EMT and stemness states, as well as the identification of novel and effective targets to develop new therapeutic strategies and preclude relapse for patients [17]. CSCs present mesenchymal status with pluripotency characteristics through the expression of EMT-associated regulators [1]. The molecular mechanisms’ interplay between EMT and stemness cellular–biological processes are poorly understood in malignancies, and its assessment can contribute to improve therapeutic modalities and patients’ quality of life. 

*TWIST1*, as a known EMT marker in cancer, can trigger the generation of a CSC status through the overexpression of stemness markers in different types of cancers [18]. In current study, we evaluated the impact of *TWIST1* ectopic expression on different involved genes in stemness state and self-renewal capacities and determined the increased levels of *BMI1*, *CRIPTO1*, *DPPA2*, *KLF4*, and *SOX2* expression following TWIST1-enforced expression in KYSE-30 cells. These results may highlight the potential of *TWIST1* to support self-renewal capacity through modulating stem cell genes’ expression pattern in ESCC line KYSE-30. 

TWIST1, as a regulator of EMT in embryogenesis, is involved in the tumorigenesis of different cancers, including sarcomas, carcinomas, and hematological malignancies [18]. It has been shown that TWIST1 plays a role either in the EMT-induced CSC phenotype or tumor stemness-induced EMT [19]. The induced EMT by *TWIST1* can expand CSCs with self-renewal potential for the growth of secondary tumors [19]. It has also been reported that the increased expression of *TWIST1* induced the expression of stemness markers and enhanced self-renewal in human head and neck squamous cell carcinoma, ESCC, as well as cervical and breast cancers [20]. The expression of *TWIST1*, *CRIPTO1*, *SOX2*, and *MSI1* were evaluated in ESCC patients, indicating their role in tumorigenesis and tumor cell aggressiveness [13,21,22,23,24]. Intriguingly, the enhanced *TWIST1* gene expression in ESCC cell lines KYSE-30 and YM-1 resulted in the significant overexpression of *OCT4* (stem cell-associate transcription factor), *MAGEA4,* and *NY-ESO1* (testicular cancer antigens), *N-cadherin*, *Occluding*, *ZEB2*, *Fibronectin*, and *Vimentin* (EMT markers), suggesting a key relation between EMT and CSC formation in esophageal tumor cells [12,13,16]. Moreover, the reduced expression of *SNAIL* gene was observed following the enforced expression of *TWIST1* in KYSE-30 cells, suggesting a negative regulatory effect of *TWIST1* on *SNAIL* expression [14]. Based on our results and considering the possibility of a well-defined function of overexpressed stemness markers in ESCC line KYSE-30 following enforced *TWIST1* expression, it may be extrapolated that the ectopic expression of *TWIST1* induced cells to undergo the EMT process for the acquisition of a CSC population and the self-renewal ability of stem-like cells. TWIST1 has been demonstrated to be involved in self-renewal through the regulation of stemness markers [4]. These results suggest that EMT induction by *TWIST1* overexpression can stimulate EMT-induced CSC state and metastasis in the tumor.

The induced expression of *BMI1* by *TWIST1* can enhance the expression of *SOX2*, *KLF4*, *NANOG*, *NF-κB*, *MRP1*, and *TERT* [4]. *NANOG* is also able to regulate the expression of *BMI1*, *TWIST1,* and *SNAIL1* to promote EMT, invasion, migration, the induction of stemness markers, metastasis, and tumor-initiating ability in breast, colon, non-small cell lung cancer (NSCLS), and head and neck squamous cell carcinoma (HNSCC) via promoter occupancy [25,26]. The physical interplay between *BMI1* and *TWIST1* in the E-cadherin promoter can lead to EMT activation and develop the CSCs subpopulation [4]. Therefore, the upregulation of *BMI1* via the enforced expression of *TWIST1* can suppress the expression of *E-cadherin* and induce proliferation, self-renewal, and chemoresistance [27]. The corporation between *BMI1* and *TWIST1* in hypoxia can induce the expression of *OCT4* and *CD44* [28]. The activation of the WNT pathway and the inhibition of tumor suppressor genes (TSGs) through *BMI1* can facilitate tumor invasion and progression [29,30]. Consequently, there is an interdependent relationship between the expression of *BMI1* and *TWIST1* in cancer initiation, differentiation, self-renewal, stemness, EMT, and CSC-mediated metastasis [4].

Our study indicated that the induction of *TWIST1* increased the expression of stemness transcription factors, including *KLF4* and *SOX2* in KYSE-30 cells, while no change in *NANOG* expression was observed. The roles of these stemness genes in tumorigenicity state have been reported previously. Numerous studies demonstrated that KLF4, SOX2, and NANOG constitute CSC markers and have key roles in sphere formation, stem cell self-renewal, cell motility, the formation and maintenance of CSC phenotype, clonogenicity and tumor regenerative ability, long-term proliferative potential of CSCs, repression of differentiation, cell cycle, migration, invasion, EMT, metastasis, and cancer progression in various malignancies [31,32]. Moreover, the overexpression of *KLF4*, *SOX2*, and *NANOG* were found in several malignancies, and their expression levels were correlated with poor prognosis, advanced-stage cancer, and shorter patient survival [31]. Different reasons may be involved in the unchanged level of *NANOG* gene expression after *TWIST1* overexpression in this study, consisting of involved transcription machinery and transcription factors, DNA-binding proteins that compete with *TWIST1*, and other regulatory proteins in the cell. Describing this situation needs further investigation.

KLF4, as an anti-proliferative factor in differentiated epithelia, play a dual function depending on tumor type, tissue, and cancer stage. While it performs as an oncogene in HNSCC, breast, skin, advanced-stage of ESCC and pancreatic cancers, it functions as a tumor suppressor gene (TSG) in lung, liver, colorectal, prostate, bladder, gastric malignancies, and high-grade dysplasia, as well as early-stage ESCCs [33,34]. Knockdown of *KLF4* and *BMI1* reduced the number and size of tumor sphere formation and tumor-initiating ability, suggesting that both *KLF4* and *BMI1* may contribute to inducing stem-like property and metastasis by TWIST1-JAGGED1-NOTCH-KLF4 signaling in HNC [35]. Evidence displayed the role of *KLF4* in triggering EMT in non-small cell lung and endometrial cancers and human nasopharyngeal carcinoma [36]. Based on previous studies and our results, *TWIST1* may induce and promote EMT through *KLF4* upregulation and its interaction with SOX2/NANOG/BMI1 in KYSE-30 cells. Similar to *KLF4*, the biological impact of *SOX2* in tumor cells is dependent on tumor type [37]. Due to the direct interaction between SOX2/NANOG/BMI1/KLF4 in the invasiveness of tumor cells, as well as its biological significance in the progression of ESCC [23], *SOX2* expression can enhance malignancy by inducing stemness properties and EMT in ESCC. It has been indicated that the ectopic expression of *SOX2* and *TWIST1* in breast cell lines MCF7 and ZR751 resulted in *SOX2*-mediated invasion/EMT via the *TWIST1*-dependent mechanism [37]. There is a direct correlation between the expression of *SOX2* and *TWIST1* in glioblastoma cells, suggesting their interaction to maintain stemness and EMT ability [38]. Interestingly, the upregulation of *SOX2* and *OCT4* can suppress *E-cadherin* expression during the reprogramming of fibroblasts and activate *SNAIL1* expression and the EMT process through the suppression of TGF-β signaling [39]. Since the transcription activity of *TWIST1* and *SOX2* in ESCs and breast cancer cells can be modulated through their binding to the promoter region [37], we hypothesized that a similar scenario may happen in ESCC. We showed that the increased expression of *TWIST1* led to the increased expression of *SOX2* in KYSE-30 cells, which may promote the invasiveness of the cells.

*CRIPTO1*, as a pluripotential ES marker, is a linker between EMT and tumor-initiating cells (or CSCs) and regulates self-renewal and tumorigenicity in cancers [40]. The upregulation of *CRIPTO1* was significantly associated with poor prognosis of patients, self-renewal, aggressive phenotypes, and tumorigenesis in esophageal and renal malignancies [21,41,42,43]. Moreover, the knockdown of *CRIPTO1* expression significantly reduced the expression of *NANOG*, *SOX2*, and *OCT4* and consequently reduced the stemness properties of ESCC cells, while its high expression was correlated with EMT, invasion, and metastasis of ESCC [41]. *TWIST1* and *CRIPTO1* co-expression was correlated with larger tumor size, advanced stages of tumor progression, poorly differentiated state of tumors, presence of distant metastases, poor 3-year survival rates, and disease progression in non-small cell lung carcinoma [44]. Herein we presented the linkage between *TWIST1* and *CRIPTO1* in KYSE-30 cells. This report is in line with previous studies describing the regulatory role for *CRIPTO1* in EMT, as an early step of invasion and metastasis, in epithelial cancer cells, including KYSE-30, EC109, and TE-1 ESCC cell lines.

Cancer cells can resemble the undifferentiated state with stem cell properties, including self-renewal ability, invasiveness, unrestricted proliferation through stem cell-associate transcription factors and germ cell or cancer/testis genes [45]. There is a transcriptional linkage between *DPPA2*, as a pluripotency-related oncogene, with the pluripotency genes of *NANOG*, *SOX2*, *OCT4*, and *SALL4*, leading to the rearrangement of the epigenome during reprogramming [46,47]. Moreover, cancer testis antigens, such as the MAGE family (especially *MAGE-A4*), *LAGE*, and *NY-ESO1,* are particularly expressed in *DPPA2*-positive NSCLC tumors [48]. There is a significant correlation between the mRNA expression of *MAGEA4* and *TWIST1* in ESCC [13]. Therefore, there may exist a correlation between the co-expression of *DPPA2* and *TWIST1* in ESCC. Herein, we elucidated that the enforced expression of *TWIST1* led to the upregulation of *DPPA2.* Consistent with the reports [12,13,14,16,21,22,48,49,50,51,52], our data also confirmed the correlation between the expression of cancer testis antigens, EMT markers, and pluripotency genes in ESCC, although further investigations are needed to determine the exact governing molecular mechanism.

Since traditional cancer treatment modalities, including surgical resection, chemo- and radiotherapies, are not sufficient to inhibit tumor relapse, *TWIST1* and other EMT transcription factors, as well as stemness state regulators, which are involved in tumor resistance against traditional therapeutic modalities, are key to the future study of cancer diagnosis and therapy. Therefore, novel therapeutic strategies focused on targeting CSCs and their molecular regulators can eradicate the CSCs and therapy resistance. Our results confirmed the potential of *TWIST1* as a proper therapeutic target for cancer treatment to inhibit EMT progress and the cancer stem-cell-like phenotype through its regulatory role of CSC markers’ gene expression, expanding insight into the *TWIST1* biology in ESCC and paving the road to an efficient targeted therapy. A combination of traditional therapeutic modalities with *TWIST1*-targeted therapy may target the whole tumor mass, including its cancer cells, as well as its CSCs subpopulations, and offer a promising strategy for cancer cure.

## 5. Conclusions

In summary, we showed that the enforced expression of TWIST1 can upregulate stem cell markers *BMI1*, *CRIPTO1*, *DPPA2*, *KLF4*, and *SOX2*, in ESCC line KYSE-30. These results may suggest a role for *TWIST1* in the stemness and self-renewal maintenance of ESCC cells and provide clues to the molecular pathway controlling the EMT-induced stemness state in cancer cells.

## Figures and Tables

**Table 1:** Primer sequences used in real-time PCR.

Gene	Primer Sequence	Annealing T, °C	Amplicon Size (bp)
BMI1	F: CGTGTATTGTTCGTTACCTGGAGAC R: CATTGGCAGCATCAGCAGAAGG	63	204
CRIPTO1	F: GGGATACAGCACAGTAAGGAG R: ACGGTGGTAGTTGTCGAGTC	61	295
DPPA2	F: AGAAATACAATCCAGGTCATCTACTTC R: GCATATCTTGCCGTTGTTCAGG	62	237
KLF4	F: TCTTCTCTTCGTTGACTTTG R: GCCAGCGGTTATTCGG	55	210
NANOG	F: GGCAATGGTGTGACGCAGAAGGC R:GCTCCAGGTTGAATTGTTCCAGGTC	65	137
MSI1	F: TGAGCAGTTTGGGAAGGTG R: TCACACACTTTCTCCACGATG	62	117
SOX2	F: AACAGCCCGGACCGCGTCAA R: TCGCAGCCGCTTAGCCTCGT	62	189
TWIST1	F: GGAGTCCGCAGTCTTACGAG R: TCTGGAGGACCTGGTAGAGG	58	201
GAPDH	F: GGAAGGTGAAGGTCGGAGTCA R: GTCATTGATGGCAACAATATCCACT	60	101

**Table 2:** The number and positions of E-box hexanucleotide consensus sequence (CANNTG) in *BMI1* transcription unit. The asterisks indicate exonic E-boxes.

**Sequence**	**Number**	**Positions**
CACTTG	4	1294-99, 1731-36, 1825-30, 4914-19
CAGGTG		
CAAGTG	6	1125-30, 1942-47, 6084-89, 8852-57
CATCTG	2	6458-63, 6811-16
CAGCTG	1	5220-25
CACCTG	1	5394-99 *
CATTTG	8	1356-61, 1668-73, 2111-16, 2495-2500, 3853-58, 7739-44, 7809-14, 10114-19
CATATG	1	1715-20
CAGATG	3	6641-46, 6888-93 *, 9107-12
CAGTTG	5	1334-39, 2046-51, 3434-39-4183-88, 4620-25
CAAATG	5	1936-41, 5040-45, 6279-75, 6614-19, 8194-99
CACATG	3	998-03-3923-28, 5774-79
CAACTG	3	2476-81-2611-16, 9243-48,
CATGTG	4	2971-76, 7219-24, 8530-35, 8597-8602
CAATTG	1	9351-56

**Table 3:** The number and positions of E-box hexanucleotide consensus sequence (CANNTG) in *Cripto1* transcription unit. The asterisks indicate exonic E-boxes.

Sequence	Number	Positions
CAGGTG	2	4940-45, 6478-83
CAAGTG	2	6447-52, 7838-43 *
CATCTG	4	2312-17, 5186-91, 6687-92 *, 7493-98 *
CAGCTG	1	2565-70
CACCTG	11	636-41, 1493-98, 2009-14, 2604-09, 3144-49, 3542-47, 3961-66, 4048-53, 4920-25, 5442-47 *, 6103-08
CATTTG	6	2143-48, 3549-54, 4760-65 *, 6312-17, 6531-36, 6815-20 *
CATATG		
CAGATG	2	2062-2067, 6596-6601
CAAATG	2	324-329, 1339-1344
CACATG	2	2246-51, 3626-31
CAACTG		730-735, 1546-1551, 7263-7268 *
CATGTG	5	1646-1651, 1977-1982, 4274-4279, 4874-4879, 7121-71268 *
CACGTG	1	7255-60 *

**Table 4:** The number and positions of E-box hexanucleotide consensus sequence (CANNTG) in *DPPA2* transcription unit. The asterisks indicate exonic E-boxes.

Sequence	Number	Positions
CACTTG	8	1070-75, 1454-59, 4400-05, 5478-83, 5613-18, 10561-66, 16659-64, 20675-80
CAGGTG	20	4994-99, 6239-44, 6505-10, 8682-87, 9174-79-9768-73, 15373-78, 10959-64, 11852-57 *, 12504-09, 15912-17, 16955-60, 17376-81, 17665-70, 18194-99, 18530-35, 19210-15, 19551-56, 20059-64, 21256-61
CAAGTG	7	705-10, 1385-90, 7490-95, 15082-87, 19075-80, 19646-51, 22042-47
CATCTG	11	1855-60, 4637-42, 10316-21, 12695-700, 13351-56, 19349-54, 20068-73, 20688-93, 20992-97, 22177-82, 22367-72
CAGCTG	4	7701-06, 12359-64, 19869-74, 19954-59
CACCTG	14	980-85, 6429-34, 9608-13, 9777-82, 11186-91, 12911-16, 13324-29, 13786-91, 15389-94, 16617-22, 16704-09, 17385-90, 21127-32, 21272-77
CATTTG	10	1883-85, 2193-98, 2914-19, 7956-61, 8216-21, 8575-80, 12762-67, 20541-46, 21034-39, 21448-53
CATATG	1	6471-76
CAGATG	3	1980-85 *, 1440-45, 15296-301
CAGTTG	6	5328-33, 7712-17, 8349-54 *, 13242-47, 13293-98, 22140-45
CAAATG	6	2501-06, 3792-97, 3907-12 *, 7203-08 *, 15645-50, 16308-13, 18294-99
CACATG	5	4534-39, 8006-11, 8611-16, 12999-13004, 22124-29
CAACTG	1	15259-64
CATGTG	10	2171-76, 3457-62, 6417-22, 11499-504, 11708-13, 13915-20, 14636-41, 17135-40, 18799-804, 19970-74
CAATTG	6	2446-51, 10305-10, 11738-43, 16200-05, 16583-88, 22717-22 *
CACGTG	1	9599-604

**Table 5:** The number and positions of E-box hexanucleotide consensus sequence (CANNTG) in *KLF4* transcription unit. The asterisks indicate exonic E-boxes.

Sequence	Number	Positions
CACTTG	2	2948-53 *, 3379-84 *
CAGGTG	4	1259-64 *, 1537-42 *, 2751-56 *, 4554-59
CAGCTG	3	489-94, 1268-73 *, 1715-20 *
CACCTG	5	950-55 *, 1438-43 *, 2527-32 *, 3437-42 *, 4146-51
CAGATG	4	3152-57 *, 4611-16 *, 4923-28 *, 5401-06 *
CAAATG	3	1988-93, 4255-60, 5455-60 *
CAACTG	1	4856-61 *
CACGTG	2	557-62, 2905-10 *

**Table 6:** The number and positions of E-box hexanucleotide consensus sequence (CANNTG) in *SOX2* transcription unit. The asterisks indicate exonic E-boxes.

Sequence	Number	Positions
CAGCTG		965-70 *, 1548-53 *, 1972-77 *
CAGATG		1013-18 *
CAGTTG		1988-93 *
CAAATG		1541-46 *
CACATG		737-42 *, 920-25 *, 1313-18 *, 1382-87 *
CATGTG		1384-89 *

## Data Availability

The data presented in this study are available on reasonably request from the corresponding author.
